# Functional magnetic resonance imaging studies of acupuncture at ST36: a coordinate-based meta-analysis

**DOI:** 10.3389/fnins.2023.1180434

**Published:** 2023-06-09

**Authors:** Jinhuan Zhang, Yongfeng Liu, Zihan Li, Qingmao Hu, Xingxian Huang, Hanqing Lv, Jinping Xu, Haibo Yu

**Affiliations:** ^1^The Fourth Clinical Medical College, Guangzhou University of Chinese Medicine, Shenzhen, China; ^2^Shenzhen Traditional Chinese Medicine Hospital, Shenzhen, China; ^3^Institute of Biomedical and Health Engineering, Shenzhen Institute of Advanced Technology, Chinese Academy of Sciences, Shenzhen, China

**Keywords:** ST36, functional magnetic resonance imaging, mechanism, acupuncture, systematic review

## Abstract

**Background:**

Functional magnetic resonance imaging (fMRI) has been widely used to investigate the brain effect of acupuncture point Stomach 36 (ST36, Zusanli). However, inconsistent results have hindered our understanding of the neural mechanisms of acupuncture at ST36.

**Objective:**

To perform a meta-analysis of fMRI studies on acupuncture at ST36 to assess the brain atlas of acupuncture at ST36 from available studies.

**Method:**

Based on a preregistered protocol in PROSPERO (CRD42019119553), a large set of databases was searched up to August 9, 2021, without language restrictions. Peak coordinates were extracted from clusters that showed significant signal differences before and after acupuncture treatment. A meta-analysis was performed using seed-based d mapping with permutation of subject images (SDM-PSI), a newly improved meta-analytic method.

**Results:**

A total of 27 studies (27 ST36) were included. This meta-analysis found that ST36 could activate the left cerebellum, the bilateral Rolandic operculum, the right supramarginal gyrus, and the right cerebellum. Functional characterizations showed that acupuncture at ST36 was mainly associated with action and perception.

**Conclusion:**

Our results provide a brain atlas for acupuncture at ST36, which, besides offering a better understanding of the underlying neural mechanisms, also provides the possibility of future precision therapies.

## 1. Introduction

Acupuncture is an ancient Chinese treatment that involves inserting needles into specific acupuncture points to treat disease and has been practiced in East Asian countries for over 2000 years. A recent review of the high-quality evidence-based medical literature reveals that acupuncture could improve the functional communication of patients with post-stroke aphasia, relieve neck and shoulder pain and non-specific lower back pain, reduce the severity of vascular dementia symptoms, and improve the nasal symptoms of allergic rhinitis (Lu et al., 2022). Currently, acupuncture is widely used around the world, with particular attention being paid to its mechanisms. However, a combination of acupoints has often been used, which makes it difficult to understand the role of specific acupoints. Therefore, it is necessary to understand the mechanisms of individual acupoints for individualized treatment. Stomach 36 (ST36, Zusanli), located 3 cm below the knee joint on the anterior portion of the leg, is a commonly used acupoint and is widely used in clinical practice. Although many animal studies have been conducted to explore the mechanisms of ST36 (Zhang et al., 2018; Lu et al., 2019), the acupoints of animals and humans are different from one another. Thus, it is necessary to further understand its function based on human acupoints.

Functional magnetic resonance imaging (fMRI) provides an experimental window to observe the human brain (Buxton, 2013). It is not only completely noninvasive but also offers excellent temporal and spatial resolution and improved sensitivity to detect task activation in individual subjects through signal averaging (Detre and Wang, 2002). Indeed, the goal of functional neuroimaging is to map the activity of the living brain in space and time. In the last three decades, a great deal of research has been performed on the application of fMRI to acupuncture (Wu et al., 1999; Kang et al., 2013; Xiao et al., 2018; Cao et al., 2020). However, due to the high cost and time-consuming nature of fMRI scanning, the sample size of each study is small. In addition, the fMRI results for acupuncture at ST36 were heterogeneous.

Therefore, it is necessary to investigate the brain effect of acupuncture at ST36 based on previously published studies in a quantitative way. We first applied seed-based d mapping with permutation of subject images (SDM-PSI), a new generation algorithm for coordinate-based meta-analysis (CBMA), to determine the most prominent and replicable brain areas of the included acupoints. Then, we investigated the functional characterizations and co-activation patterns of significant clusters using behavioral domains, paradigm classes, and meta-analytic connectivity modeling (MACM) analysis, respectively.

## 2. Materials and methods

The review process followed the preferred reporting items for systematic reviews and meta-analyses (PRISMA) statement (Moher et al., 2009), and the meta-analysis protocol was registered with PROSPERO^1^ (registration number: CRD42020204050).

### 2.1. Selection procedure

#### 2.1.1. Search strategies

Studies of acupuncture on some acupoints were searched from PubMed, Web of Science, Wanfang, and the Chinese National Knowledge Infrastructure (CNKI) from the inception of the databases up to August 2021 to identify relevant studies. The following search terms were used: (“acupuncture” or “acupuncture therapy” or “electroacupuncture” or “EA”) and (“zusanli” or “ST36”) and (“functional magnetic resonance imaging” or “fMRI” or “BOLD” or “ReHo” or “ALFF” or “fALFF”) NOT (“mice” or “rat” or “animal”). Additional articles were identified by cross-referencing the reference lists of the included articles.

#### 2.1.2. Selection criteria

In this review, we included all studies that used fMRI to investigate the effect of acupuncture on the human brain. To be included in this meta-analysis, research had to meet the following inclusion criteria: (1) it had to include healthy subjects only; (2) it had to include verum acupuncture only or verum versus sham acupuncture only using task-based fMRI with the whole-brain acquisition; (3) it only needed to report ST36; (4) it reported findings in 3D coordinates in the Montreal Neurological Institute (Evans et al., 1993) or Talairach space (Talairach and Tournoux, 1988); (5) it concerned manual acupuncture or electroacupuncture. Studies were excluded if they: (1) examined needling stimulation with tasks, such as finger movement; (2) investigated only region of interest (ROI) results or used functional connectivity, independent component analysis, and graph theory analytical methods; (3) Consisted of reviews, case reports, conferences, abstracts, and animal studies; (4) only included fMRI results between acupuncture and sham acupuncture; (5) had no effective value.

### 2.2. Data extraction

Two researchers manually extracted basic information and the peak voxel coordinates of the included studies, such as first author, year of publication, gender, mean age, number of subjects, field strength, analysis methods, stereoscopic template, and statistical threshold. Any discrepancies were discussed with the third researcher until a consensus was reached.

### 2.3. Meta-analysis procedure

This meta-analysis was performed using SDM-PSI version 6.21 (Albajes-Eizagirre et al., 2019). Detailed SDM methods have been described on this website.^2^ First, text files of peak coordinates and effect sizes (e.g., *t*-values) of fMRI differences between post-treatment and pre-treatment were extracted. During the preprocessing, the lower and upper bounds of the possible effect-size values of the studies were recreated using the above files. Then, the mean map was generated by voxel-wise calculation of the random-effects mean of the study maps, weighted by the sample size, intra-study variability, and between-study heterogeneity. Family-wise error (FWE) correction (*p* < 0.05 and voxel extent ≥10) using the threshold-free cluster enhancement approach (TFCE) and 5,000 permutations was initially used.

Peak coordinate values were extracted for heterogeneity statistics and publication bias analyses. Heterogeneity between studies was assessed using the I2 statistic in a random-effects model, with *I*^2^ < 50% indicating low heterogeneity (Higgins et al., 2003).

Publication bias was assessed using funnel plots and Egger’s tests (Egger et al., 1997). An asymmetric plot and *p* < 0.05 were considered statistically significant.

Pain-related brain regions were searched,^3^ then common brain regions of activation of ST36 and pain-related brain regions were extracted.

Finally, co-activation pattern analysis and functional characterization were performed as described in detail in a previous study (Zhang et al., 2021).

## 3. Results

### 3.1. Included studies and sample characteristics

**Figure 1:**
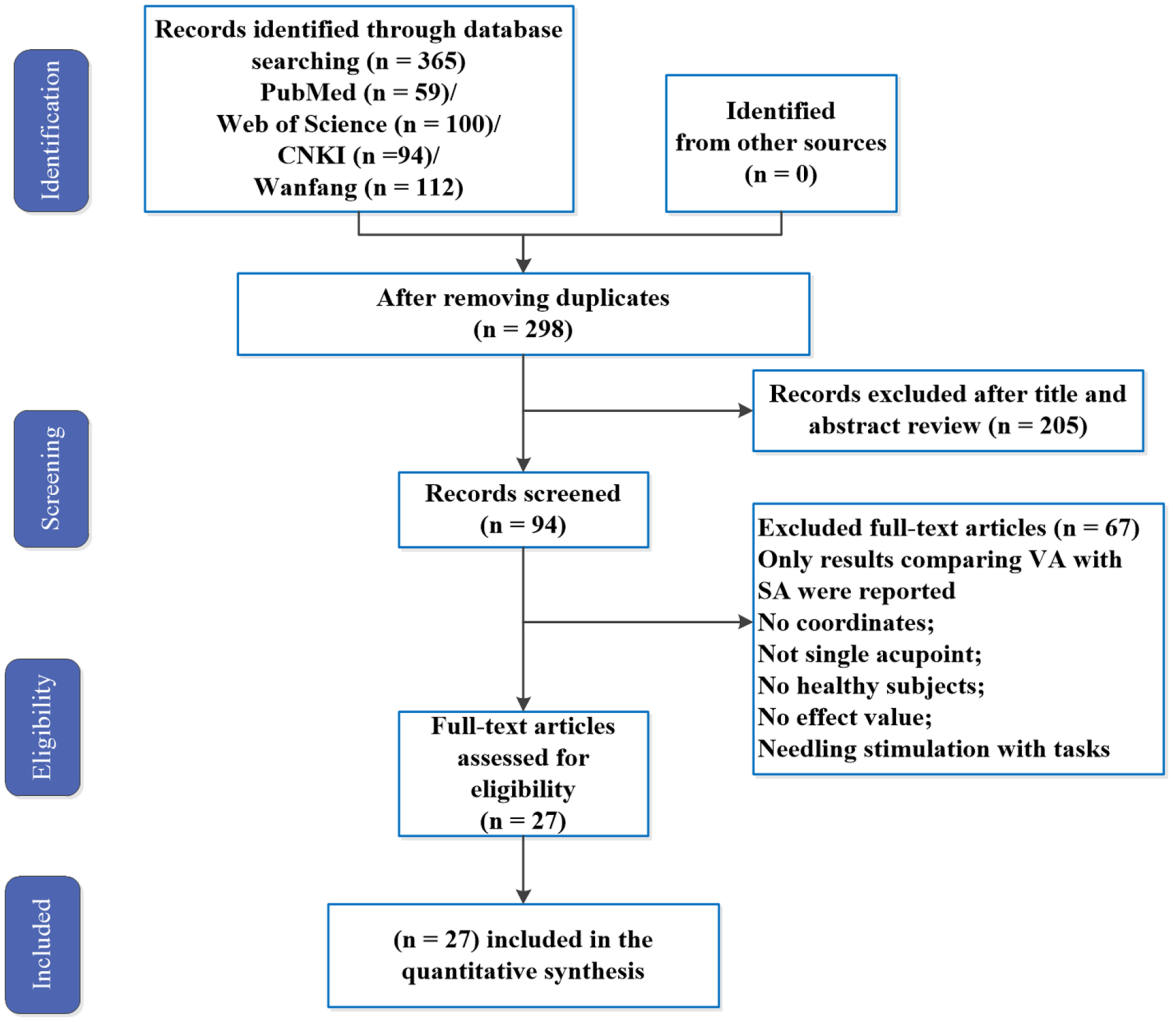
PRISMA flow diagram. VA, verum acupuncture; SA, sham acupuncture.

**Figure 2:**
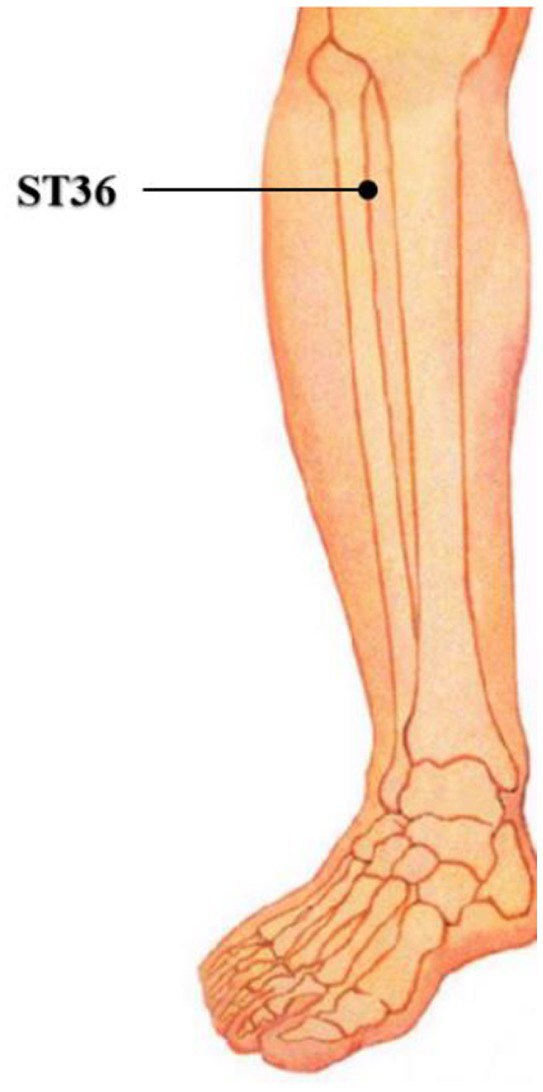
ST36 location.

A total of 27 studies met the inclusion criteria for the meta-analysis. The study selection process is shown in Figure 1. A total of 542 HC subjects reporting after vs. before contrast brain response, including ST36 (27), are shown in Table 1 and Figure 2.

**Table 1:** Demographic and clinical characteristics of the included ST36 studies.

Number	Author, year	Gender (M/F)	Age (mean)	Field strength	Software	Statistical threshold	Template
1	Fang et al. (2005)	13	19–31	1.5T	AFNI	*p* < 0.001	Tal
2	Hui et al. (2005)	11	29.8 ± 7.5	1.5T	AFNI	*p* < 0.001	Tal
3	Napadow et al. (2005)	13	21–42	3T	AFNI	*p* < 0.005	Tal
4	Jiang (2006)	10 M/5 F	20–33	1.5T	SPM2	*p* < 0.05, corrected	Tal
5	Wu et al. (2007)	5 M/6 F	23–39	1.5T	SPM2	*p* < 0.05, corrected	Tala
6	Zhang et al. (2007)	5 M/6 F	23–27	1.5	SPM2	*p* < 0.01	Tal
7	Bai et al. (2009)	16	21.4 ± 1.8	3T	SPM 5	*p* < 0.005, uncorrected	Tal
8	Wang (2009)	6 M/6 F	25.2 ± 1.44	1.5T	AFNi	*p* < 0.005	Tal
9	Long et al. (2009)	7 M/10 F	24. 6 ± 0. 3	1.5T	SPM5	*p* < 0.001, uncorrected	MNI
10	Jiang et al. (2010)	18 M/14F	23.8 ± 4.3	1.5T	SPM2	*p* < 0.05	Tal
11	Feng et al. (2011)	6 M/6F	25.2 ± 1.44	1.5T	AFNI	*p* < 0.005	Tal
12	Wu et al. (2011)	8 M/8F	25 ± 3.3	1.5T	SPM5	*p* < 0.05	Tal
13	Claunch et al. (2012)	46	2.88	1.5T	AFNI	*p* < 0.003, uncorrected	Tal
14	Duan et al. (2012)	8 M/8 F	25.0 ± 3.3	1.5T	SPM5	*p* < 0.05	Tal
15	Liu (2012)	10 M/11 F	21–29	1.5T	SPM8	*p* < 0.01, uncorrected	Tal
16	Jiang et al. (2012)	10 M/5F	25 ± 4. 5	1.5T	SPM2	*p* < 0.001	Tal
17	Sun et al. (2012)	50	23.3 ± 2.1	3T	SPM5	*p* < 0.0001, uncorrected	Tal
18	Fu et al. (2013)	5 M/5 F	23 ~ 25	1.5T	SPM8	*p* < 0.05	MNI
19	Jiang et al. (2013)	18	19 to 27	3T	SPM5	*p* < 0.05, FDR	MNI
20	Li et al. (2013)	21 M/24 F	18–25	3T	SPM8	*p* < 0.005	MNI
21	Dong et al. (2013)	8 M/8F	19–25	3.0T	SPM8	*p* < 0.001, uncorrected	MNI
22	Jin et al. (2014)	20	22–25	3T	SPM5	*p* < 0.0001	Tal
23	Li C. et al. (2014)	18 M/16 F	25.0 ± 2.3	1.5T	/	/	Tal
24	Tian et al. (2014)	30	29.0 ± 7.8	1.5T	SPSS 13.0	*p* < 0.05	Tal
25	Zhong et al. (2014)	6 M/6 F	21 ± 2.6	1.5T	SPM5	*p* < 0.05	Tal
26	Liu et al. (2014)	4 M/6 F	20–34	3.0T	SPM8	*p* < 0.05, FDR	MNI
27	Nierhaus et al. (2015)	20	19–31	3T	SPM 8	*p* < 0.05	Tal

### 3.2. Primary meta-analytic results

**Figure 3:**
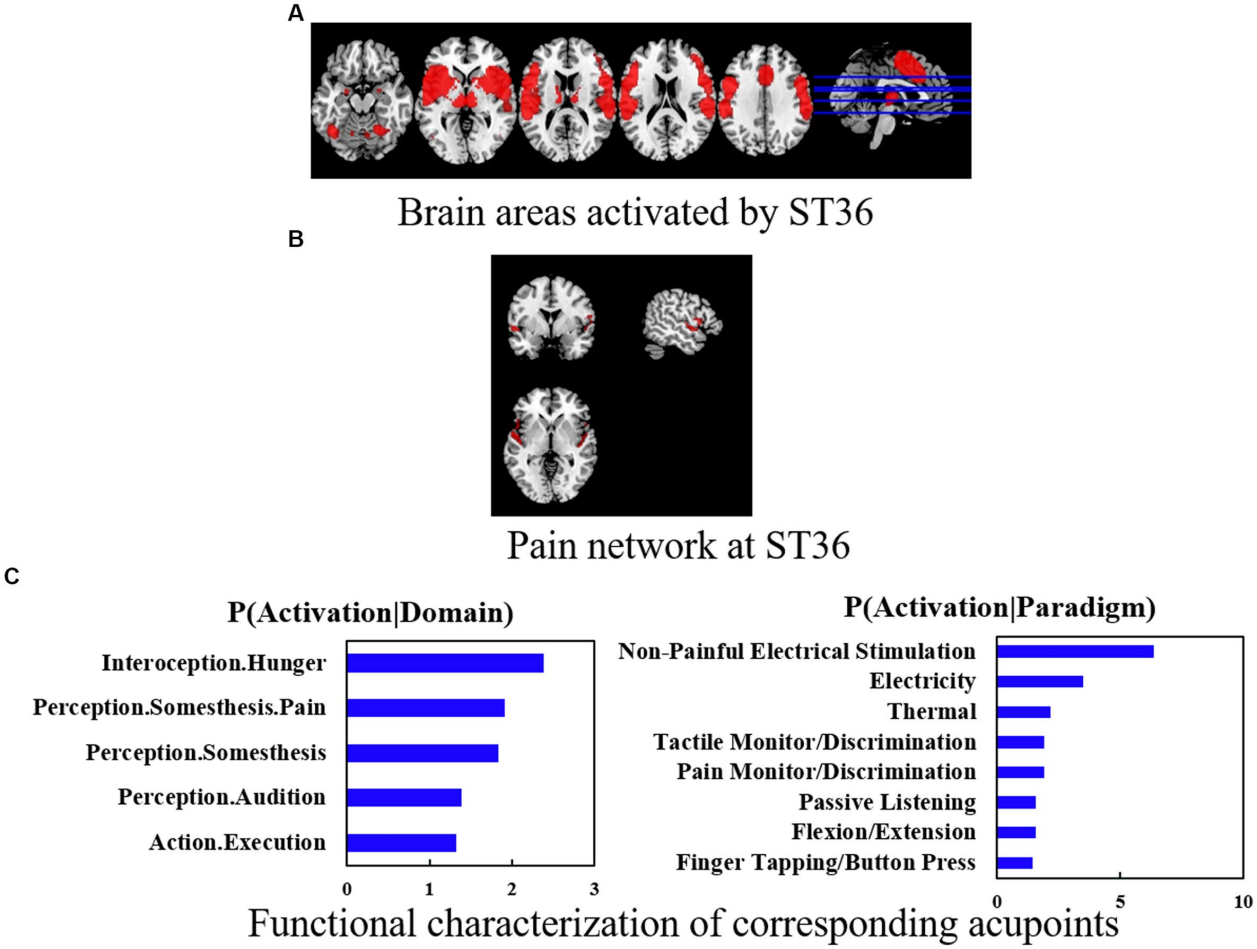
**(A)** Brain areas activated by ST36. **(B)** Pain network at ST36. **(C)** Functional characterization of corresponding acupoints. Coactivation connectivity of significance levels was thresholded at *p* < 0.05, corrected for multiple comparisons with FDR. Functional characterization of significance levels was thresholded at *p* < 0.05, corrected for multiple comparisons using FWE, clustering threshold at voxel-level *p* < 0.001.

The ST36 group showed hyperactivation in the bilateral cerebellum, hemispheric lobule VIII, bilateral Rolandic operculum (ROL), and right supramarginal gyrus (SMG.R). The results of the SDM analysis are summarized in Table 2 and Figure 3A.

**Table 2:** Brain regions activated by acupuncture stimulation at ST36 (compared to resting state).

MNI coordinate	SDM-Z	*p*	Voxels	Description	*I*^2^ (%)
−14, −80, −50	4.703	<0.001	2,353	Left cerebellum, hemispheric lobule VIII	0
−56, 0, 4	4.82	<0.001	854	Left rolandic operculum, BA 48	0
58, 8, 8	4.019	<0.001	795	Right rolandic operculum, BA 6	0
64, −20, 36	4.85	<0.001	637	Right supramarginal gyrus, BA 1	0
34, −62, −52	4.24	<0.05	288	Right cerebellum, hemispheric lobule VIII	0

TFCE Corrected *p* < 0.05.

In terms of heterogeneity, *I*^2^ (0%) showed little heterogeneity in between-study variability on peak coordinates of ST36. In addition, Egger’s tests indicated no significant publication bias on the peak coordinates of ST36.

In terms of co-activation patterns and functional characterization, the bilateral cerebral, fusiform, postcentral, and temporal areas were co-activated with ST36 (Figures 3A,B). Functional characterization showed that the ST36 was mainly associated with action and perception. The PCs showed similar results (Figure 3C).

### 3.3. Pain and specific networks

We investigated the pain network and non-pain network activation brains of ST36 and found that ST36 can activate pain-related networks including the left cerebellum, bilateral Rolandic operculum, left cerebellum crus 1, right supramarginal, and right postcentral. The specific-related network was also found in several brain areas like the right inferior frontal gyrus (IFG.R.), left superior temporal gyrus (STG.L), right cerebellum 8 (cerebellum 8R), and left cerebellum crus 2 (cerebellum crus 2L) (Table 3 and Figure 3B).

**Table 3:** Pain network and non-pain network at ST36.

ST36	MNI coordinates	*Z*	Voxels	Description
Pain network	−6, −72, −44	4.1561	196	Left cerebellum 8
	−56, 0, 4	4.8204	1,365	Left Rolandic operculum
	58, 8, 8	4.019	872	Right Rolandic operculum
	64, −20, 36	4.8504	898	Right supramarginal
Non-pain network	56, −20,40	3.8086	190	Right postcentral
	54, 14, 4	3.5035	298	Right inferior frontal gyrus, opercular part
	−54, 0, 0	4.6475	475	Left superior temporal gyrus
	34, −62, −52	4.2399	464	Right cerebellum 8
	−14, −80, −50	4.7032	2,539	Left cerebellum Crus 2

### 3.4. Functional characteristics

Functional characterization showed that acupuncture at ST36 was mainly associated with motor, cognition, learning and memory, self-awareness, and other aspects (Figure 3C).

## 4. Discussion

In this study, we systematically reviewed task-based fMRI studies of acupuncture at ST36 in healthy subjects. First, we obtained the results of the brain regions activated by acupuncture at ST36 and then distinguished between pain-overlapping activation areas and non-pain activation areas based on the brain areas activated by acupuncture at ST36, where the pain-overlapping brain regions are also the pain sensations produced by the acupuncture itself, and the non-pain activation brain regions are also the specific function of acupuncture at ST36.

### 4.1. Activation patterns and functional characteristics

In this study, we found that bilateral cerebellum, hemispheric lobule VIII, SMG.R, bilateral Rolandic operculum (ROL), and right anterior thalamic projections were activated by acupuncture at ST36. However, this is inconsistent with a previous meta-analysis of ST36 (Zhang et al., 2019). The reasons for this could be explained by the following two points: first, the previous review investigated the effect of deqi when acupuncture at ST36 was applied on brain function; second, different statistical methods and thresholds may be available, this study is more rigorous in this regard.

Evidence suggests that the primary characteristic roles of the cerebellum not only include motor control function but also its non-motor function in cognitive control and learning processing (Moulton et al., 2010; Ruscheweyh et al., 2014; Moberget and Ivry, 2016). Although the cerebellum has often been shown to respond to painful stimuli, the current findings show that the cerebellum is more concerned with visceral than somatic pain (Claassen et al., 2020), which is consistent with the fact that ST36 is often used clinically to treat visceral pain (Fan et al., 2021). In addition, recent studies have also shown that electroacupuncture at ST36 can modulate cerebellar lobule VIII to treat comorbid brain regions of depression and pain (Lottering and Lin, 2021), further confirming ST36’s important role in the treatment of these conditions.

Studies also demonstrate that the right SMG.R has implications for cognitive functions such as temporal perception and attention, pitch memory performance (Wiener et al., 2010), emotion recognition (Wada et al., 2021), visual word recognition (Stoeckel et al., 2009), and episodic memory encoding (Rubinstein et al., 2021).

The Rolandic operculum processes integrated exteroceptive-interoceptive signals that are necessary for interoceptive awareness and bodily self-consciousness (Blefari et al., 2017). Several studies have reported that the Rolandic operculum not only plays a role in emotion processing but also in the sensory system for gustatory and visceral sensations together with the cingulate-operculum network (Eickhoff et al., 2006; Lelic et al., 2015). The gastrointestinal tract has been reported to be sensitive to emotion (depression, anxiety, and stress); the connection between the brain and gastrointestinal organs is explained by the theory of a gut-brain axis (top-down and bottom-up) (Kim and Shin, 2017).

Therefore, based on the functional characteristics of acupuncture at ST36, we found that this may be effective in the treatment of motor, cognition, learning and memory, self-awareness, and other aspects. Importantly, in clinical studies, ST36 is also often used for the treatment of gastrointestinal diseases and to improve constipation (Lu et al., 2019; Wu et al., 2020), pain (Zuo et al., 2019; Wan et al., 2021), cognition (Li Q. Q. et al., 2015; Yang et al., 2020), and other related diseases.

### 4.2. Pain-specific overlapping activation patterns

Acupuncture needles are applied to the body, which not only produces the sensation of pain but also has therapeutic effects. However, it is not clear whether this effect overlaps with the areas of the brain where the pain is generated or whether acupuncture is specific.

In this study, we found that the cerebellum, insula, superior temporal gyrus, and postcentral regions of the brain were implicated in the pain network, which is consistent with previous research by Chae et al. (2013) showing that acupuncture stimulation is associated with multidimensional pain.

In addition, many previous fMRI studies (Hui et al., 2000; Kong et al., 2007; Fang et al., 2009) have described the activation of these sensorimotor brain areas as a common feature of acupunctural stimulation. Thus, activation in sensorimotor brain areas such as the insula, thalamus, SI, and SII presumably reflects the involvement of the sensory pain-associated components of acupunctural needle stimulation. One previous meta-analysis suggested that brain response of acupuncture needle stimulation involved in sensory, cognitive, and affective dimensions of pain (Chae et al., 2013). Another study also demonstrated that regardless of the type of harmful stimulus, some brain regions such as the thalamus, insula, and ACC have a significant potential for activation, suggesting that acupuncture, as an external stimulation, could activate some important brain regions (Duerden and Albanese, 2013).

Importantly, we found that a specific-related network of acupuncture on ST36 was found in several brain areas, including IFG.R, STG.L, cerebellum 8R, and cerebellum crus 2L, which is consistent with a recent systematic review similar to this one that showed the opercular part of IFG.R, STG.L, and right median cingulate/paracingulate gyri (MCG.R) regions were positively activated following ST36 acupuncture (Huang et al., 2022).

The IFG.R has a role in modulating cognitive control in several domains, such as inhibitory control and the process of allocating attention (Or-Borichev et al., 2023). It also plays a causal role in the control of interference in memory retrieval (Stramaccia et al., 2017). STG.L plays an important role in auditory processing and social cognition (Bigler et al., 2007). The cerebellum is crucially involved in a wide spectrum of cognitive and affective functions (Jacobi et al., 2021), in addition to its well-established role in balance control and motor function (Chen et al., 2022). According to the above information, we could conclude that cognitive function is a common characteristic of specific activation patterns of ST36, which is consistent with previous research findings (Lu et al., 2014; Li F. et al., 2015). We speculate that acupuncture at ST36 could not only improve cognitive function but also regulate movement, auditory processing, and so on.

Although we found some interesting results from the current data, the interpretation of the results is complex because the central stimulation is not just a linear association. Therefore, it is difficult for us to determine the diseases that could be treated by acupuncture at ST36 through the activated brain region of acupuncture at ST36, so it needs to be combined with clinical practice to finally determine the brain map of acupuncture at ST36. Nonetheless, it still offers us some clinical guidance. In addition, although ST36 acupuncture produces pain-related brain regions linked to the physical stimulation of acupuncture, these cannot be ruled out as part of the treatment.

### 4.3. Limitations

Our study has several limitations. First, the studies included in our meta-analysis differ with respect to experimental design, analytical methods, and software, which may lead to some heterogeneity. However, we did not find any heterogeneity in this study. Second, a previous study (Li Z. J. et al., 2014) found that the disease state is an important factor in the cerebral response to acupuncture stimulation. In this review, we only paid attention to healthy subjects, due to the small number of studies and sample size of acupuncture for patients. Third, the lack of control over the design of sham acupuncture reduces the reliability of the conclusions. Finally, due to the small number of included studies, the results need to be interpreted with caution.

### 4.4. Suggestions for future studies

Future studies should increase the sample size because a sufficient one is necessary to ensure sample power and obtain stable and reliable results.

In terms of brain response, the difference between verum acupoints and sham acupoints should be investigated in more studies to confirm the specificity of acupoints. In addition, the detailed acupoint operation of ST36 should be clearly described, and future acupuncture clinical trials should follow the STRICTA standards (MacPherson et al., 2002).

In terms of fMRI, there is some room for improvement, such as higher resolution and strict statistical analysis to obtain reliable results. Specifically, an appropriate experimental design for investigating acupuncture effects, such as a block- and event-related design, is crucial. Recently, a data-driven approach was considered suitable for describing response characteristics. Perhaps the most suitable method is to define the ROI to explore the efficacy of acupuncture based on the abnormal brain area of the corresponding disease. Finally, a strict threshold is also one of the important conditions to obtain reliable results. Furthermore, a checklist for the fMRI study should be followed in order to provide some reference for further research (Poldrack et al., 2008).

## 5. Conclusion

In conclusion, fMRI is a useful visualization tool for investigating the central mechanisms of acupoints. This study found that acupuncture at ST36 activated the bilateral cerebellum, hemispheric lobule VIII, bilateral Rolandic operculum, and right supramarginal gyrus, suggesting that acupuncture at ST36 could be used to treat motor, cognitive, learning and memory, and emotion-related disorders. This study provides new insights into the therapeutic mechanisms of acupuncture. In the future, it will be possible to map the brain altas of each acupoint, allowing it to achieve precise treatment of diseases.

## Author contributions

HY, JX, and QH designed the whole study. JZ and YL analyzed the data. JZ wrote the manuscript. ZL searched and selected the studies. XH and HL participated in the discussion. All authors contributed to the article and approved the submitted version.

## Funding

This work was supported by the National Natural Science Foundation of China (grant nos. 62006220 and 62001462), and the Shenzhen Science and Technology Research Program (grant nos. JCYJ20180507182441903, JCYJ20200109114816594 and JCYJ20210324111206017).

## Conflict of interest

The authors declare that the research was conducted in the absence of any commercial or financial relationships that could be construed as a potential conflict of interest.

## Publisher’s note

All claims expressed in this article are solely those of the authors and do not necessarily represent those of their affiliated organizations, or those of the publisher, the editors and the reviewers. Any product that may be evaluated in this article, or claim that may be made by its manufacturer, is not guaranteed or endorsed by the publisher.

## Footnotes

^1^ http://www.crd.york.ac.uk/PROSPERO

^2^ https://www.sdmproject.com/

^3^ https://neurosynth.org/
